# Human papillomavirus 18 E6 inhibits phosphorylation of p53 expressed in HeLa cells

**DOI:** 10.1186/2045-3701-2-2

**Published:** 2012-01-13

**Authors:** Amrendra K Ajay, Avtar S Meena, Manoj K Bhat

**Affiliations:** 1 National Centre for Cell Science, NCCS Complex, Pune University Campus, Ganeshkhind, Pune - 411007, India; 2 Renal Division, Brigham and Women's Hospital, Harvard Medical School, Boston, MA, USA

**Keywords:** HPV, p53, phosphorylation, cervical cancer, E6

## Abstract

**Background:**

In HPV infected cells p53 function is abrogated by E6 and even ectopically expressed p53 is unable to perform tumor suppressor functions. In addition to facilitating its degradation, E6 may also inhibit p53 transactivity, though the mechanisms are still poorly understood. It has been reported that inhibition of p300, an acetyltransferase responsible for p53 acetylation is inactivated by E6. Activation of overexpressed p53 to cause cell growth inhibition is facilitated by its phosphorylation. Previously, we reported that non-genotoxically overexpressed p53 in HeLa cells needs to be phosphorylated to perform its cell growth inhibitory functions. Since over expressed p53 by itself was not activated, we hypothesized an inhibitory role for E6.

**Results:**

Majority of reports proposes E6 mediated degradation of p53 as a possible reason for its inactivation. However, results presented here for the first time demonstrate that overexpressed p53 is not directly associated with E6 and therefore free, yet it is not functionally active in HPV positive cells. Also, the stability of overexpressed p53 does not seem to be an issue because inhibition of proteasomal degradation did not increase the half-life of overexpressed p53, which is more than endogenous p53. However, inhibition of proteasomal degradation prevents the degradation of endogenous p53. These findings suggest that overexpressed p53 and endogenous p53 are differentially subjected to proteasomal degradation and the reasons for this discrepancy remain unclear. Our studies demonstrate that p53 over expression has no effect on anchorage independent cell-growth and E6 nullifies its cell growth inhibitory effect. E6 overexpression abrogates OA induced p53 occupancy on the p21 promoter and cell death as well. E6 did not decrease p53 protein but phospho-p53 level was significantly reduced.

**Conclusion:**

We report for the first time that E6 de-activates p53 by inhibiting its phosphorylation. This prevents p53 binding to p21 promoter and thereby restraining its cell-growth inhibitory functions. Our study provides new evidence indicating that viral protein E6 inhibits p53 transactivity by mechanism independent of degradation pathway.

## Background

Approximately 470,000 new cases of cervical cancer are diagnosed every year and close to 230,000 women worldwide die, with the majority (~80%) of incidence occurring in developing countries. Human papillomavirus (HPV) infection is the main causative agent for cervical cancer. Reports suggest that 99.7% of cervical cancers harbor integrated HPV DNA in host cell genome [1]. HPV presence is reported in 5-11% of oral cancers [2]. In head and neck cancers the percentage of HPV infection is low and it accounts for 11-25% [3-8]. In developed countries 50-70% of oropharyngeal and tonsillar carcinomas are associated with HPV infection [9,10]. Papillomaviruses are also reported to be present in colon and 90% of anal cancers [11-14]. HPVs are classified in two categories, low risk, which has less or no potential and high risk, which has potential to cause carcinogenesis. HPV 16 and 18 are high risk HPVs, accounting for more than 50% of cervical cancers and are considered as a major cause of other (head and neck as well as anal) cancers too.

The two onco-proteins of HPV, E6 and E7 cause transformation, immortalization and promote carcinogenesis primarily by binding to important tumor suppressor's p53 and pRb, thereby completely deregulating cell cycle checkpoints [15-18]. E6 and E7 alone can also immortalize, deregulate cell cycle and cause transformation of even primary cultures [19-21]. E6 degrades p53 by E3 ubiquitin dependent and independent proteasomal degradation [18,22].E6 also inhibits p53 transactivity by inhibiting acetylation [23], because of its ability to bind directly and degrade p300, an important acetyltransferase [24,25]. To completely abrogate p53 activity E6 also degrades bax, a major p53 downstream apoptosis inducer [26]. It has been reported that inhibition of E6 by its specific siRNA reactivates dormant p53 pathways, and the mechanisms by which functions are restored are not clear [27-29].

Activation of many proteins is accomplished by phosphorylation, which is caused by group of enzymes called kinases [30]. Concomitantly, activated proteins are kept under check by phosphatases, thus opposing the effects of kinases [31]. p53 being a phospho-protein is trans-activated by phosphorylation and deactivated by dephosphorylation [32]. Okadaic acid (OA), a specific inhibitor of protein phosphatases, promotes phosphorylation of p53 or its upstream kinases at various residues [33,34]. Recently, we reported that OA activates overexpressed p53, causing cell cycle arrest and apoptosis in HeLa cells [35]. Very little is known about the phosphorylation status of p53 in response to its silencing by E6, except for one study which reported that p53 is phosphorylated at multiple residues by transiently transfected E6 [36]. In the present investigation we demonstrate that upon OA treatment overexpressed p53 is phosphorylated at serine 46 residue and ectopic expression of E6 promotes its dephosphorylation. Our study provides new evidence indicating that viral protein E6 inhibits p53 transactivity by mechanism independent of degradation pathway.

## Results

### Over expression of p53 in HeLa cells

**Figure 1:**
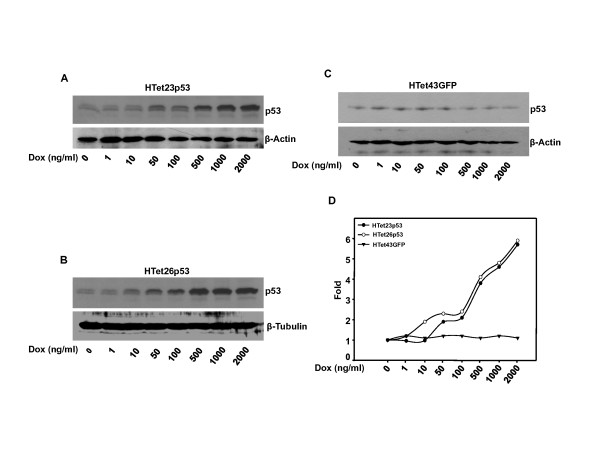
**p53 expression is regulated in a dose dependent manner by Dox**. (A and B) Cells treated with Dox for 48 h were harvested and western blot for p53 was performed. (C) No induction of p53 in response to Dox was observed in HTet43GFP cells. (D) Fold induction of p53 was calculated by densitometric analysis of p53 western blot in HTet23p53, HTet26p53 and HTet43GFP cells taking control = 1with normalization to β-Actin or β -Tubulin.

Earlier we reported the development of HeLa cells in which p53 is conditionally overexpressed and induction of p53 is tightly regulated by doxycycline (DOX) in a dose dependent manner. Two p53 expressing clones (HTet23p53 and HTet26p53) and one GFP expressing clone (HTet43GFP) were used in this study [35]. Dox in a dose dependent manner induces p53 protein in both HTet23p53 (Figure 1A) and HTet26p53 (Figure 1B) cells as compared to HTet43GFP cells (Figure 1C). Densitometric analysis of bands in these blots suggests that in comparison with non-induced state, more than 5 fold increases in p53 level was achieved in the presence of 2000 ng/ml Dox (Figure 1D). No alteration in p53 level was detected in HTet43GFP cells (Figure 1C) cells under identical experimental conditions.

### p53 is expressed in a time dependent manner

**Figure 2:**
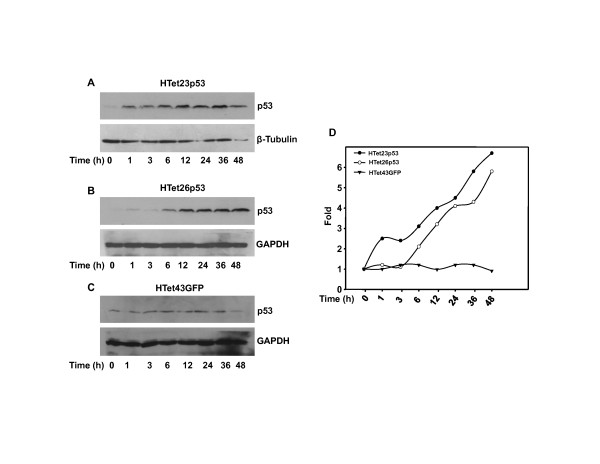
**p53 expression is regulated in a time dependent manner**. (A, B and C) HTet23p53, HTet26p53 and HTet43GFP cells were treated with 1000 ng/ml Dox for indicated time points and western blots were performed for p53. (D) Fold induction was calculated by densitometric analysis of p53 in HTet23p53, HTet26p53 and HTet43GFP cells taking 0 h = 1 with normalization to β -Tubulin or GAPDH.

To study the kinetics of p53 expression single dose (1000 ng/ml) of Dox was added for different time durations (1 h to 48 h) and western blot was performed. As shown in Figure 2, p53 expression was initiated within 1 h of Dox addition and it increased progressively up to 48 h of incubation in HTet23p53 (Figure 2A) and HTet26p53 cells (Figure 2B). No changes in basal p53 levels were detected in HTet43GFP (Figure 2C) cells under identical experimental conditions. At 48 h, 5-fold increase in p53 protein was detected in HTet23p53 and HTet26p53 cells as compared to HTet43GFP cells (Figure 2D).

### Overexpressed p53 has no effect on anchorage independent growth

**Figure 3:**
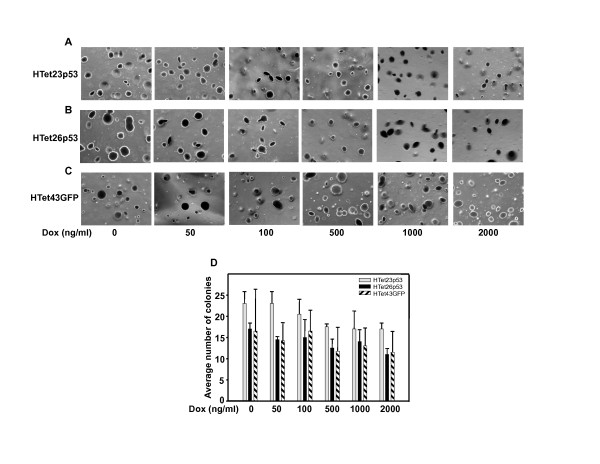
**p53 over expression did not reduce colony number**. Five thousand cells were plated in low-melting agarose containing complete medium and allowed to grow for 30 days. (A, B and C) Cells were stained with crystal violet and photographs were taken under microscope for HTet23p53, HTet26p53 and HTet43GFP plates. (D) Colonies were counted and average number of colonies was plotted vs Dox concentration. Bar represents average of colonies from two representative fields (± SE).

The tumorigenic potential of p53 overexpressing cells was assessed by soft agarose assay, which is often used method for characterization of cellular growth properties. In HTet23p53 (Figure 3A) as well as HTet26p53 (Figure 3B) cells; no decrease in anchorage independent growth was detected even after inducing p53 with upto 2000 ng/ml Dox. HTet43GFP cells served as control (Figure 3C). No significant difference in the colony number and size of p53 overexpressing clones as compared to GFP expressing clone was visible. Dox treatment slightly decreased colony number in all the three cell lines, which may be because of long term Dox cytotoxicity.

### Overexpressed p53 is stable

**Figure 4:**
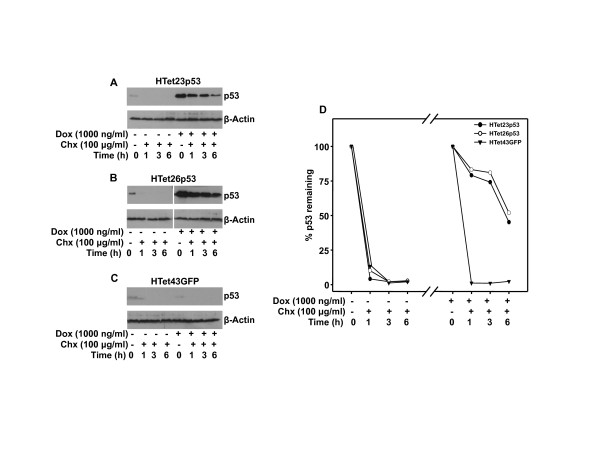
**Overexpressed p53 is stable**. (A and B) p53 was overexpressed with 1000 ng/ml of Dox for 48 h or not overexpressed and western blotswere performed after indicated time of Chx treatment in HTet23p53 and HTet26p53 cells. (C) Western blot for p53 was performed with or without addition of 1000 ng/ml Dox for 48 h followed by Chx treatment for indicated time points in HTet43GFP cells.(D) Graphical representation of percentage of p53 protein remaining after indicated time points in HTet23p53 and HTet26p53 cells by densitometric analysis following normalization with β-Actin. Protein percentage for 0 h Chx was taken as 100.

To perform tumor suppressor functions p53 stability is essential. Therefore to ascertain that overexpressed p53 is stable and it is not degraded by E6, cycloheximide (Chx) chase experiment was performed. Cells were treated with Chx for different time points to inhibit protein synthesis and then western blotted to detect p53. In the presence of Chx over expressed p53 was present in significant amount in HTet23p53 and HTet26p53 cells (Figure 4A and 4B) even after 6 h. Under these experimental conditions endogenous p53 in same cells (Figure 4A and 4B) or in HTet43GFP cells (Figure 4C) decreased to undetectable levels just within 1 h. The half-life of overexpressed p53 was calculated to be 6 h as compared to that of less than 1 h for endogenous p53 (Figure 4D).

### Inhibition of proteasome promotes stability of endogenous p53 but not for ectopically overexpressed p53

**Figure 5:**
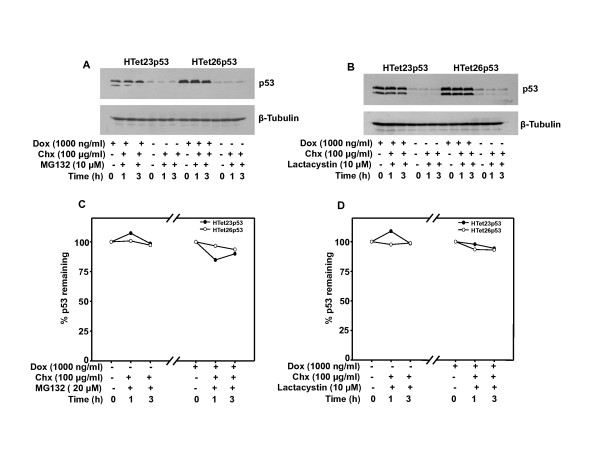
**Overexpressed p53 is not stabilized by inhibition of proteasomal degradation**. Cells were treated with proteasomal inhibitors (MG132 or Lactacystin) 1 h prior to Dox addition and incubated for 48 h. Thereafter, cells were treated with Chx for indicated time points and western blots were performed. (A) MG132 treatment in HTet23p53 and HTet26p53 cells and p53 protein expression. (B) Lactacystin treatment in HTet23p53 and HTet26p53 cells and p53 protein expression.

It is very well known that p53 undergoes proteasomal degradation. Inhibiting proteasomal degradation by two specific inhibitors MG132 and lactacystin did not cause accumulation of overexpressed p53 protein in HTet23p53 as well as HTet26p53 cells upto 3 h. Treatment with these inhibitors prevents degradation of endogenous p53 protein in HTet23p53, HTet26p53 and HTet43GFP cells even after Chx chase for 1 h (Figure 5A and 5B). Taken together these results suggest that endogenousp53 is stabilized in the presence of inhibitors whereas overexpreseed p53 levels are not altered.

### Inhibition of protein phosphatase 2A promotes cell death and E6 reverses it by inhibiting promoter occupancy of activated overexpressed p53

**Figure 6:**
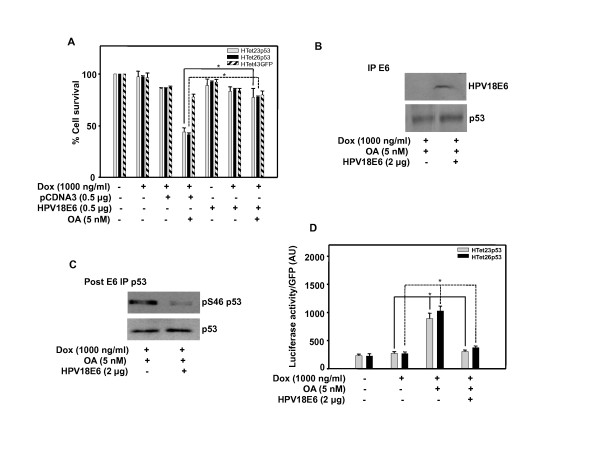
**Overexpressed p53 is functionally impaired by HPV E6**. (A) HTet23p53, HTet26p53 or HTet43GFP cells were transfected with vector or HPV18 E6 plasmid and 18 h post transfection cells were treated with OA 1 h prior to Dox addition. MTT assay was performed after 48 h. Bar represents variations among the wells of an experiment done twice in triplicate. * Indicates P < 0.01 (B) HTet26p53 cells were transfected with HPV18 E6 plasmid and treated as mentioned in A and immunoprecipitation was performed first with E6 antibody (first IP) and secondly by p53 antibody. p53 or E6 was detected in immunoprecipitated complex. (C) p53 post-IP (second IP) following E6 IP was performed and p53 or phospho-p53detection by western blotting was performed. (D) Cells were transfected with p21luciferase construct with or without HPV18 E6 plasmid and treated as mentioned in A. Luciferase assay was performed and luciferase/GFP reading was plotted. Bar represents results from an experiment done in triplicate. (± SE). * Indicates P < 0.05.

To determine whether E6 inhibits activity, p53 was first activated by 5 nM OA, a protein phosphatase 2A (PP2A) inhibitor. As a consequence of p53 activation cell growth is retarded in p53 overexpressing HTet23p53 and HTet26p53 cells compared to p53 non-overexpressing HTet23p53, HTet26p53 or HTet43GFP cells. Cell survival inhibitory effect was abolished by ectopic expression of HPV 18 E6 in OA treated p53 overexpressing cells (Figure 6A). Overexpressed p53 is not completely associated with E6 and ectopic expression of E6 does not further enhance this association, though p53 is detected in co-immunoprecipitated complex (Figure 6B). To confirm the presence of free p53 in the lysate immunoprecipitated by E6 antibody (first IP), second immunoprecipitation with p53 (FL-393) was done. Interestingly, ectopic expression of E6 did not affect the p53 protein but it does drastically decrease the level of Ser46 phosphorylated p53 (pSer46p53) (Figure 6C). Further, to confirm that E6 mediated inhibition of p53 phosphorylation actually is responsible for cell growth inhibition, we performed luciferase reporter activation assay for well-known transcriptional target p21. p21 promoter was activated by OA treatment and E6 over expression inhibits promoter activation (Figure 6D).

### Overexpression of p53 causes cell death and E6 expression promotes cell survival in p53/E6 null lung carcinoma cell line

**Figure 7:**
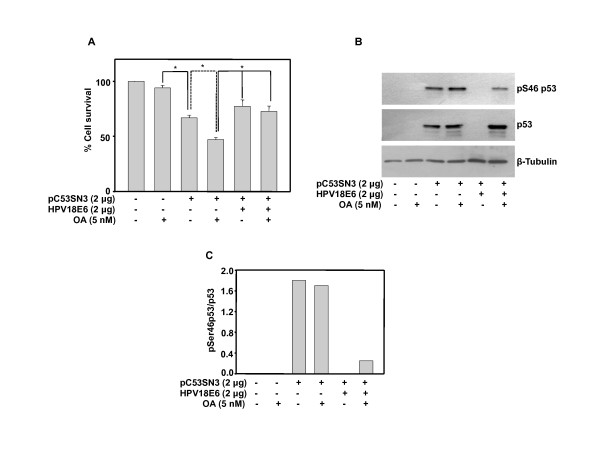
**Overexpressed p53 is active and is made non-functional by HPV 18 E6 in H1299, a p53 and E6 null cell line**. (A) H1299 cells were transfected with pC53-SN3 and/or HPV18 E6 plasmids and 18 h post transfection cells were treated with OA. MTT assay was performed after 48 h. Bar represents variations among the wells of an experiment done twice in triplicate. * Indicates P < 0.01 (B) H1299 cells were transfected with pC53-SN3 and/or HPV18E6 plasmids and 18 h post transfection cells were treated with OA for 48 h and western blot was performed for pSer46p53 and p53.(C) Densitometric analysis was performed by normalization with p53 and ratio of pSer53 and p53 was plotted.

To investigate the consequences of direct functional interaction between p53 and E6 we transfected wild type-p53 with or without E6 in H1299 lung carcinoma cells, which are null for p53, and E6. p53 overexpression decreases cell survival by 25 percent confirming it being active in non HPV cells. OA enhances activity of p53 and therefore cell survival decreases further by additional 25 percent. Co-expression of HPV18E6 in p53 expressing cells treated with or without OA promotes survival (Figure 7A). At protein level, as expected no p53 is detected in these cells and it is expressed in cells transfected with p53 plasmid. Interestingly, co-transfection with HPV18E6 construct reduces p53 levels below detection in non OA treated cells, whereas in OA treated cells p53 is detected. Reduction of p53 level (Figure 7B, Lane 5) may be due to its degradation by E6. Comparatively, the protein levels are higher in OA treated cells with or without E6. OA treatment enhances pSer46p53 levels (Figure 7B, Lane 4) and E6 diminishes OA promoted phosphorylation (Figure 7B, Lane 6). Also, phosphorylated p53 levels decrease in the presence of E6 (Figure 7B, Lane 6).

## Discussion

p53 overexpression in HeLa cells does not exhibit cell growth inhibitory functions whereas in non HPV positive H1299 cells it causes cell death (unpublished observation). This difference in p53 activity is likely to be dependent on the appropriate post-translational modifications including phosphorylation status, which depends on host cell phenotype. Also, E6 overexpression has differential effect on p53 protein stability. In HeLa cells it does not degrade p53 but in H1299 it completely degrades it.

Others and we reported that activation of overexpressed p53 to cause cell growth inhibition is facilitated by its phosphorylation at Ser46 [35-37]. Majority of reports suggest the E6 or E7 mediated degradation/functional impairment of p53 as possible reason for its inactivation [38-40]. However, for the first time results presented here demonstrate that overexpressed p53 is not directly associated with E6 and therefore free, yet it is not functionally active in HPV positive cells. Also, overexpressed p53 has significantly increased half life and proteasomal inhibitors do not exhibit any detectable impact on the levels of overexpressed p53. These results suggest that it is likely p53 activation might be inhibited by yet unknown mechanism.

Ectopic expression of E6 does not decrease overexpressed p53 protein level in HeLa and H1299 cells treated with OA, which could be because of swamping out of the available E6 and/or E6AP. This proposition derives support from a report describing the involvement of an E6 associated protein (E6 AP), which forms a ternary complex essential for ubiquitination of p53 [41]. It is also possible that the post-translational modification as well as conformation of overexpressed p53 might be different and is therefore not recognizable by the E6 and E6AP complex. The involvement of specific post-translational alterations is consistent with our [35] and other reports [37,42]. Together, these results suggest that expressed p53 is activated only in the presence of PP2A inhibitor and p53 phosphorylation at a key residue is very critical for specific DNA binding as well as promoter selection under various stress conditions. Interestingly, p53 phosphorylation is diminished by over expression of E6 in HeLa cells (Figure 6C), which indicates that E6 causes inactivation of p53 by inhibiting its phosphorylation. Also, E6 expression significantly inhibits p21 promoter occupancy of the overexpressed and activated p53, which has an impact on cell growth (Figure 6D and 6A). Further, to confirm this hypothesis we utilized p53 and E6 null H1299 cells. Overexpression of p53 causes cell death (Figure 7A), which parallels with p53 and pSer46p53 levels. OA treatment further enhances cell killing by stabilizing p53 and pSer46p53 levels. Interestingly, E6 coexpression causes complete degradation of p53 in non-OA treated cells whereas in OA treated cells E6 does not reduce p53 levels though it decreases pSer46p53 level (Figure 7B), which correlates with increased cell survival under these condition (Figure 7A). Future studies with small molecule activator like PRIMA that activates mutant p53, [43] will be useful in delineating the mechanisms of p53 activation.

## Conclusion

The results presented here provide insight into differential regulation of endogenous and exogenous p53 and the role HPV E6 plays in its phosphorylation and activation. These findings imply that replacement of degraded, mutated p53 protein or functionally inactivated p53 with the wild-type one will have significant therapeutic importance only when its activation is also achieved simultaneously. The functionality of p53 depends on the cellular background.

## Materials and methods

### Chemicals and antibodies

Doxycycline (Dox) and cycloheximide was purchased from Sigma (St. Louis, MO). Tet-system approved serum and Hygromycin solution (50 mg/ml) was purchased from BD (Mountain View, CA). G418 was purchased from (USB, OH). Antibodies against p53 (FL-393 goat polyclonal; DO1-HRP conjugated mouse monoclonal), E6 (goat polyclonal and mouse monoclonal), GAPDH (goat polyclonal), β-Tubulin (rabbit polyclonal) and β-Actin (goat polyclonal) were purchased from Santa Cruz Biotechnology (Santa Cruz, CA). MG132 and Lactacystin were purchased from Calbiochem (CA). Okadaic acid (OA) was purchased from Invitrogen Corporation. Phospho-p53 Ser46 antibody (rabbit polyclonal) was purchased from Cell Signaling Technology (Danvers, MA). HRP conjugated secondary antibodies were purchased from Santa Cruz Biotechnology.

### Cell lines

HeLa and H1299 cell lines were purchased from American Type Culture Collection (Manassas, VA) and maintained in our in-house National Cell Repository, National Centre for Cell Science (NCCS), Pune, India. Dox inducible cell lines were developed by utilizing BD-TetOn system and stably transfecting with pTetOn and pTREp53 or pBIEGFP. Dulbecco's minimum essential medium (DMEM) was purchased from Invitrogen Corporation. The inducible cell lines were regularly cultured in DMEM, supplemented with 10% Tet system approved fetal bovine serum at 37°C with 5% CO_2_. Inducible cell lines were maintained in 100 μg/ml of G418 and 50 μg/ml of hygromycin.

### Plasmids and transfection

pTet-On, pTRE, pTK-Hyg and pBIEGFP were purchased from BD. pC53-SN3 (p53 plasmid) was a kind gift from Dr. Bert Vogelstein, John Hopkins, Baltimore, MD USA. p53 fragment of pC53-SN3 was sub-cloned in *BamH1 *site of pTRE and was renamed as pTREp53. p21 luciferase was kind gift from Dr. Bert Vogelstein. pFLAG-HPV 18 E6 plasmid was kind gift from Dr. McCance DJ, University of Rochester, USA.

Transfections were performed by Lipofectamine2000 (Invitrogen) as per manufacture's protocols.

### Soft agarose assay

Culture dish was layered with 1 ml of 0.7% agarose. Five thousand HTet23p53, HTet26p53 and HTet43GFP cells were plated in 0.5% low melting agarose (FMC Bioproducts, ME) containing DMEM and 10% FBS with or without Dox and incubated after layering with 1 ml complete medium. Medium containing indicated concentration of Dox was changed every 4^th ^day. After 30 days plates were stained with 0.1% crystal violet for 1 h and photographed under a microscope. Colonies of more than 50 cells were counted and graph was plotted from the average of two independent fields from each plates.

### Cell proliferation assay

Cell proliferation was determined by methylthiazole tetrazolium (MTT) assay. Cells were seeded at the density of 7,500 per well into 96 well plates and allowed to adhere for 24 h. Cells were transfected with pCDNA3 or HPV 18 E6 plasmid construct by Lipofectamine2000 reagent. Eighteen hour post transfection cells were washed thrice with DMEM and treated with Dox in the presence of absence of OA and further incubated for 48 h. Fifty microliter of MTT (1 mg/ml) was added to each well and incubated for 4 h at 37°C. Hundred microliter of 2-propanol was added and incubated in shaking condition at room temperature for 10 min. Absorbance was taken at 570 nm using 630 nm as reference filter. Absorbance given by untreated cells was considered as 100% cell survival.

### Preparation of whole cell lysate and western blotting

Following indicated treatments, cells were washed thrice with ice-cold phosphate buffered saline (PBS) and lysed in ice-cold lysis buffer (50 mM Tris-Cl, pH 7.5, with 120 mM NaCl, 10 mM NaF, 10 mM sodium pyrophosphate, 2 mM EDTA, 1 mM Na_3_VO_4_, 1 mM PMSF, 1% NP-40 and protease inhibitor cocktail (Roche Diagnostics, Germany). Equal amount of protein was resolved on SDS-PAGE and western blotting was preformed as described earlier [44]. Where ever possible blots were stripped by incubating the membranes at 50°C for 30 min in stripping buffer (62.5 mM Tris-Cl pH 6.7, 100 mM mercaptoethanol, 2% SDS) with intermittent shaking. Membranes were washed thoroughly with TBS and reprobed with required antibodies. Otherwise gels run in duplicates were probed for the desired proteins by western blotting and then compiled.

### Cycloheximide chase assay

HTet23p53, HTet26p53 and HTet43GFP cells grown in a 35 mm plate were treated with 1000 ng/ml of Dox for 48 h and then 100 μg/ml cycloheximide (Chx) was added. Cells were further incubated for indicated time points and processed for western blotting. For inhibitor experiments cells were treated with MG132 or Lactacystin 10 μM each 48 h prior to Chx addition and harvested after indicated time points after Chx addition.

### Immunoprecipitation

After indicated treatment cells were harvested and lysed in RIPA buffer. Equal amount of protein (400 μg) was taken and lysates were pre-cleared with 50 μl protein A/G plus agarose (Invitrogen Corporation) for 30 min. Agarose beads were pelleted and supernatant was incubated with anti-E6 goat polyclonal antibody overnight at 4°C in an IP rotator. Fifty microliter protein A/G plus agarose was then added in antibody-antigen complex with gentle shaking for 4-5 hours at 4°C (first IP). The immune complex bound to protein A/G plus agarose was separated by centrifugation at 4000 rpm and supernatant was immunoprecipitated with anti-p53 goat polyclonal antibody and as described above (second IP). Target as well as its associated proteins was disrupted from protein A/G plus agarose beads by adding SDS gel sample buffer, resolved on SDS PAGE and processed for western blotting [44] with mouse monoclonal E6 and p53 antibodies.

### Luciferase assay

Semi-confluent HTet23p53 and HTet26p53 cells plated in a 12 well plate were co- transfected with pEGFPC1 and p21 luciferase plasmids by Lipofectamine2000. Eighteen hour post-transfection 1000 ng/ml Dox was added with or without OA and further incubated for 48 h. Luciferase assay was performed as per manufacturer's protocol (Amersham Biosciences). GFP reading was taken as an internal control for normalization of transfection efficiency and graphs were plotted.

### Statistical Analysis

Data are expressed as the mean of three independent experiments. Statistical comparisons are made using two tailed students paired t-test by assuming variance is unequal (SPSS Inc, USA) and *P *value < 0.05 was considered as significant.

## List of Abbreviations

Dox: doxycycline; HPV: human papillomavirus; OA: okadaic acid; PP2A: protein phosphatase 2A.

## Competing interests

The authors declare that they have no competing interests.

## Authors' contributions

AKA and MKB designed the study. AKA performed experiments in HeLa cells and analyzed the data. ASM performed experiment with H1299 cells. AKA and MKB wrote the manuscript and MKB supervised the project. All authors read and approved the final manuscript.
